# TDP-43 expression in mouse models of amyotrophic lateral sclerosis and spinal muscular atrophy

**DOI:** 10.1186/1471-2202-9-104

**Published:** 2008-10-28

**Authors:** Bradley J Turner, Dirk Bäumer, Nicholas J Parkinson, Jakub Scaber, Olaf Ansorge, Kevin Talbot

**Affiliations:** 1 University of Oxford, MRC Functional Genetics Unit, Department of Physiology, Anatomy and Genetics, South Parks Road, Oxford, OX1 3QX, UK; 2 Department of Neuropathology, John Radcliffe Hospital, Headley Way, Oxford, OX3 9DU, UK; 3 Department of Clinical Neurology, John Radcliffe Hospital, Headley Way, Oxford, OX3 9DU, UK

## Abstract

**Background:**

Redistribution of nuclear TAR DNA binding protein 43 (TDP-43) to the cytoplasm and ubiquitinated inclusions of spinal motor neurons and glial cells is characteristic of amyotrophic lateral sclerosis (ALS) pathology. Recent evidence suggests that TDP-43 pathology is common to sporadic ALS and familial ALS without SOD1 mutation, but not SOD1-related fALS cases. Furthermore, it remains unclear whether TDP-43 abnormalities occur in non-ALS forms of motor neuron disease. Here, we characterise TDP-43 localisation, expression levels and post-translational modifications in mouse models of ALS and spinal muscular atrophy (SMA).

**Results:**

TDP-43 mislocalisation to ubiquitinated inclusions or cytoplasm was notably lacking in anterior horn cells from transgenic mutant SOD1^G93A ^mice. In addition, abnormally phosphorylated or truncated TDP-43 species were not detected in fractionated ALS mouse spinal cord or brain. Despite partial colocalisation of TDP-43 with SMN, depletion of SMN- and coilin-positive Cajal bodies in motor neurons of affected SMA mice did not alter nuclear TDP-43 distribution, expression or biochemistry in spinal cords.

**Conclusion:**

These results emphasise that TDP-43 pathology characteristic of human sporadic ALS is not a core component of the neurodegenerative mechanisms caused by SOD1 mutation or SMN deficiency in mouse models of ALS and SMA, respectively.

## Background

Spinal muscular atrophy (SMA) and amyotrophic lateral sclerosis (ALS) are the commonest forms of human motor neuron disease in children and adults, respectively. The disorders share the feature of vulnerability of lower motor neurons in the anterior horn of the spinal cord, implicating possible common factors in motor neuron degeneration. ALS also affects upper motor neurons in the cerebral cortex, and a proportion of cases demonstrate more widespread changes that overlap pathologically and clinically with frontotemporal lobar degeneration (FTLD). While SMA is an autosomal recessive genetic disorder caused by deletions of the survival motor neuron 1 (SMN1) gene with resulting SMN deficiency [1], only about 5–10% of ALS cases are familial (fALS) [2,3]. Dominant mutations in the superoxide dismutase 1 gene (SOD1) cause approximately 20% of the familial cases, and thus contribute the largest single group of hereditary ALS. The genetic contribution of single genes or at risk haplotypes to the majority of sporadic cases is currently thought to be modest [4]. Novel insights into the aetiopathogenesis of ALS have come from the discovery of the TAR DNA binding protein (TDP-43) as a major constituent of the characteristic ubiquitinated inclusions found in neuronal and glial cells in ALS [5]. While ubiquitination of proteins is no proof of their pathogenicity, the role of TDP-43 as a disease causative protein is suggested by the observation that, in affected cells, TDP-43 is absent from its normal location in the nucleus and redistributed to the cytoplasm, where it displays various staining patterns from diffuse distribution to strict co-localisation in ubiquitinated aggregates [6,7]. In addition to the abnormal TDP-43 distribution, biochemical analysis of disease tissue reveals a characteristic disease signature of TDP-43 in urea soluble protein extracts, characterised on Western blots by high molecular weight species, 25 kD C-terminal fragments and 45 kD hyperphosphorylated protein bands [5]. Further confirmation of the pathogenic role of TDP-43 comes from a series of publications reporting mutations in TARDBP in both familial and sporadic ALS. An increasing number of mutations have been described which predominantly affect the conserved C-terminal glycine-rich domain of TDP-43 predicting abnormal RNA or protein interactions. Furthermore, the existence of *TARDBP *mutations in autosomal dominant ALS with demonstrable TDP-43 pathology strengthens the evidence for a causal role of TDP-43 in inherited forms of motor neuron disease. [8-12].

At present, it is unclear whether the pathogenic effect of TDP-43 results from the formation of toxic aggregates, or from the loss of its nuclear function. It is noteworthy that in mammals TDP-43 has been shown to interact in the nucleus with the SMN protein, deficiency of which results in the motor neuron disease SMA [13]. SMN is present in the cytoplasm and nucleus of mammalian cells. In the latter, it forms discrete nonmembrane bound structures called 'gems' (for gemini of coiled bodies). Gems are in a complex relationship with Cajal bodies, structures characterised by the presence of coilin, and gems and Cajal bodies colocalise to varying degrees depending on the stage of development and tissue type. Motor neurons show the highest degree of colocalisation between gems and Cajal bodies [14]. If SMN levels are reduced in cells [15] or mice[16], Cajal body formation as assessed by staining with anti-coilin antibody is impaired. Given the interaction between TDP-43 and SMN in the nucleus, one hypothesis is that loss of TDP-43 from the nucleus could lead to motor neuron degeneration in ALS, in part because of an alteration in nuclear SMN function. Conversely, nothing is known about a potential role of TDP-43 in SMA.

While the pathological features of ALS described above have been replicated in cases of sporadic ALS, they are probably absent in cases of fALS caused by SOD1 mutations [17]. Sporadic and non-SOD1-related fALS, together with certain types of FTLD, can therefore be seen as TDP-43 spectrum proteinopathies, whereas SOD1 fALS may be a phenocopy of sporadic ALS with a different aetiopathogenesis [18]. This also means that the most commonly used mouse models of ALS, in which mutant SOD1 is overexpressed [19], might not accurately reflect the pathology found in the vast majority of human cases. Indeed, there is evidence that there is no TDP-43 redistribution in three mouse models of SOD1 ALS [20]. However, mouse models do not always reflect every aspect of human disease [21], and the absence of TDP-43 redistribution does not rule out the presence of abnormal biochemical TDP-43 species. In particular, while ALS in humans is clearly an age dependent disease, all aspects of ALS neuropathology might not emerge within the short lifespan of the mouse.

In the present study, we confirmed the previously described finding of absent TDP-43 redistribution to the neuronal cytoplasm in the SOD1^G93A ^model of ALS. We have extended these findings to address the question of whether TDP-43 is altered in its nuclear distribution, expression level or biochemical state. To examine a potential role of TDP-43 in SMA, we then looked for evidence of TDP-43 redistribution, altered expression or biochemistry in a commonly used model of moderate to severe SMA, the 'SMNΔ7 mouse' [22].

## Methods

### Transgenic mice

Transgenic SOD1^G93A ^mice derived from the B6SJL-TgN (SOD1-G93A) 1Gur line (Jackson Laboratory) were backcrossed onto a pure C57BL6 background and generously provided by Prof. Pamela Shaw (University of Sheffield). Mice were maintained by mating heterozygous transgenic males with C57BL6 females. Disease endpoint mice (post-natal day 120) and age-matched wild-type mice were killed by lethal injection (Pentoject, IP) in accordance with Home Office regulations. Transgenic Smn+/-;SMN2;SMNΔ7 mice ([22]) were maintained as heterozygote breeding pairs. Homoyzgote Smn-/-;SMN2;SMNΔ7 mice reached the disease endpoint at postnatal day 13 (P13) and were killed with wild-type littermates as described above. A minimum of 3 mice of each genotype were used for each procedure below. All genotypes were confirmed by routine PCR analysis using tail genomic DNA.

### Immunohistochemistry and Immunofluorescence

Mice were transcardially perfused with phosphate buffered saline (PBS) followed by 4% paraformaldehyde (PFA) in 0.1 M phosphate buffer under terminal anaesthesia with pentobarbitone. Lumbar spinal cords were dissected, post-fixed in 4% PFA overnight at 4°C and embedded in paraffin. 6 μm horizontal sections were dewaxed and autoclaved for 10 minutes at 121°C in 0.01 M citrate buffer pH 6.0 for antigen retrieval. Immunostaining was performed using overnight incubation at 4°C with mouse anti-SMN (BD Transduction lab, 1:320), rabbit anti-TDP-43 (Proteintech 1:500), rabbit anti-p80 coilin (Dundee Cell products 1:200) and rabbit anti-ubiquitin (Dako 1:500). Primary antibodies were visualised using a Dako REAL EnVision detection system (Dako UK Ltd). For immunofluorescence, sections were blocked in 4% normal goat serum, 2% BSA in PBS-T for 30 minutes prior to primary antibody incubation. Alexa Fluor 488 and 568 conjugated antibodies raised in goat were used as secondaries (1:500, Molecular Probes, Invitrogen). Nuclei were counterstained with DAPI.

### Image analysis

2 spinal cord sections were analysed for each of 3 animals of each genotype. Per section, five large alpha motor neurons were examined per ventral horn (20 per animal). Motor neurons were identified by their location in the ventral horn, large nucleus, prominent nucleolus, polygonal shape and relatively large size. Only cells with clearly visible nucleolus were chosen for analysis. Images were captured using a Zeiss LSM 510 laser scanning microscope with a 63× oil immersion objective and 2× digital zoom. Image analysis was performed using Image J software (NIH). In order to quantify nuclear TDP-43, images of motor neuron nuclei were displayed in Image J with automatic setting of brightness and contrast. All distinct speckles of nuclear TDP-43 fluorescence with a minimum diameter of 1 μm were then counted manually using the point picker function. Cajal bodies and TDP-43 speckles were counted blind to genotype.

### Sequential biochemical fractionation and immunoblotting

Brains and lumbar spinal cords were dissected, snap frozen and sequentially extracted as previously described [5]. Briefly, tissues were extracted at 200 mg/ml in low salt (LS) buffer by sonicating for 15 sec, incubating on ice for 30 min and centrifuging at 14,000 rpm for 30 min at 4°C. Pellets were sequentially extracted in high salt Triton (TX), sarkosyl (SARK) and urea (UR) buffers as above. Proteins were quantified using the reducing agent-compatible BCA assay kit (Pierce, Rockford, IL). Samples (50 μg) were electrophoresed through 12.5% SDS-polyacrylamide gels and transferred to nitrocellulose membranes. Blots were blocked with 5% (w/v) non-fat milk in TBST for 1 hr and incubated with rabbit TDP-43 (1:1,500), SOD1 (1:1,000, Calbiochem) or mouse β-actin (1:5000, Abcam) antibodies overnight at 4°C. Blots were probed with HRP-conjugated antibodies (1:10,000, Amersham) and developed with enhanced chemiluminescence reagents (Roche).

For protein dephosphorylation, urea extracts were dialysed against 50 mM Tris-HCl, pH 7.9, 100 mM NaCl, 10 mM MgCl_2 _and 1 mM DTT using Slide-A-Lyzer mini dialysis units (Pierce). Dialysates (50 μg) were treated with 10 U calf intestinal phosphatase (New England Biolabs) for 1 hr at 37°C and the reaction was terminated by addition of SDS loading buffer.

### Real-time PCR

Frontal cortex, brainstem, cerebellum and lumbar spinal cord were dissected and snap-frozen. RNA was extracted using the RNeasy kit (Qiagen) and cDNA was reversed transcribed using oligo(dT) primers and Superscript II (Invitrogen). qRT-PCR was performed using 900 nM forward 5'-TCCCCTGGAAAACAACAGAG-3' and reverse 5'-CCAGACGAGCCTTTGAGAAG-3' mouse TARDBP primers with Power SYBR Green master mix on an ABI Prism 7700 real-time PCR machine (Applied Biosystems). TARDBP levels were normalised against amplified GAPDH levels using forward 5'-TGTGACTTCAACAGCAACTC-3' and reverse 5'-GTGGACCTCATGGCCTACAT-3' primers. Fold change from wild-type mice was calculated for each tissue. Genotypes were measured in triplicate and compared with an unpaired *t*-test using GraphPad Prism 3.0 software (GraphPad).

## Results

### Ubiquitinated cytoplasmic inclusions but no TDP-43 redistribution in SOD1^G93A ^anterior horn cells

**Figure 1:**
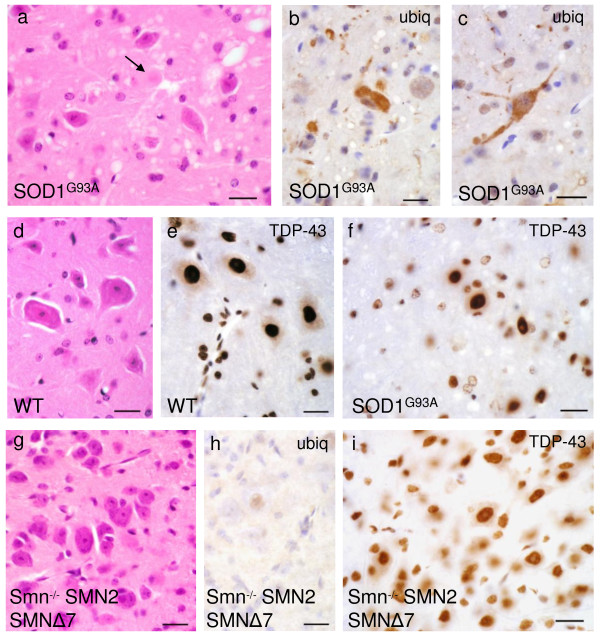
**Spinal cord sections of transgenic SOD1^G93A ^mice (a-c, f) show anterior horn cell degeneration, hyaline aggregates (arrow) and vacuolation (a) not present in corresponding wild-type (d).** Amorphous cytoplasmic aggregates are ubiquitinated and occasionally extend into proximal processes (b, c). TDP-43 remains located in the nucleus of anterior horn cells and shows no distinct aggregates (f). The appearances are essentially indistinguishable from wild-type (e) apart from possible slightly weaker TDP-43 signal in presumed reactive glia (f). Samples from transgenic Smn^-/-^;SMN2;SMNΔ7 spinal cord (g-i) show residual motor neurons (g) without ubiquitin positivity (h) and preserved nuclear TDP-43 signal (i). Scale bar in all images = 20 μm.

We first examined spinal cords of two common mouse models of motor neuron disease for ubiquitin or TDP-43 histopathology, completely screening all stained sections visually for abnormal neuronal TDP-43 localisation (figure 1). Motor neurons in terminal SOD1^G93A ^mice, but not WT animals, showed extensive cytoplasmic staining with ubiquitin antibodies (figure 1b,c). However, TDP-43 immunoreactivity was confined to nuclei only in both genotypes (figure 1e,f). Thus, in contrast to sporadic and most familial cases of human ALS, TDP-43 did not aggregate into distinct inclusions and was not mislocalised from the nucleus to the cytoplasm in anterior horn cells of SOD1^G93A ^mice. Interestingly, it appeared that some of the nuclei of presumed reactive astroglia in endstage SOD1^G93A ^mice showed a weaker TDP-43 signal than glia in WT litter mates (figures 1e,f). Neither cytoplasmic TDP-43 redistribution, nor cytoplasmic ubiquitin staining was present in spinal cords of endstage SMA animals (figure 1h,i).

### Reduction of Cajal bodies in SMA animals, but no alteration of nuclear TDP-43 architecture in SMA or SOD1^G93A ^animals

**Figure 2:**
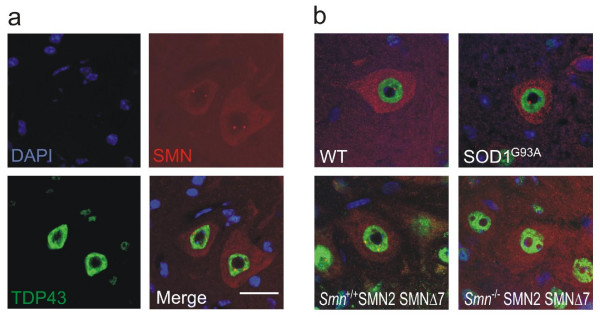
**(a) Wild-type mouse spinal cord sections stained for TDP-43 and SMN.** Large alpha motor neurons show diffuse cytoplasmic SMN stain and punctate nuclear SMN, Cajal bodies. TDP-43 shows only nuclear and no cytoplasmic staining. (b) Representative merged TDP-43 and SMN stains for all genotypes. Note absence of Cajal bodies in the SMA mouse. The pattern of TDP-43 immunoreactivity is unaltered in SOD1^G93A ^and SMA mice. Scale bar = 20 μm.

**Figure 3:**
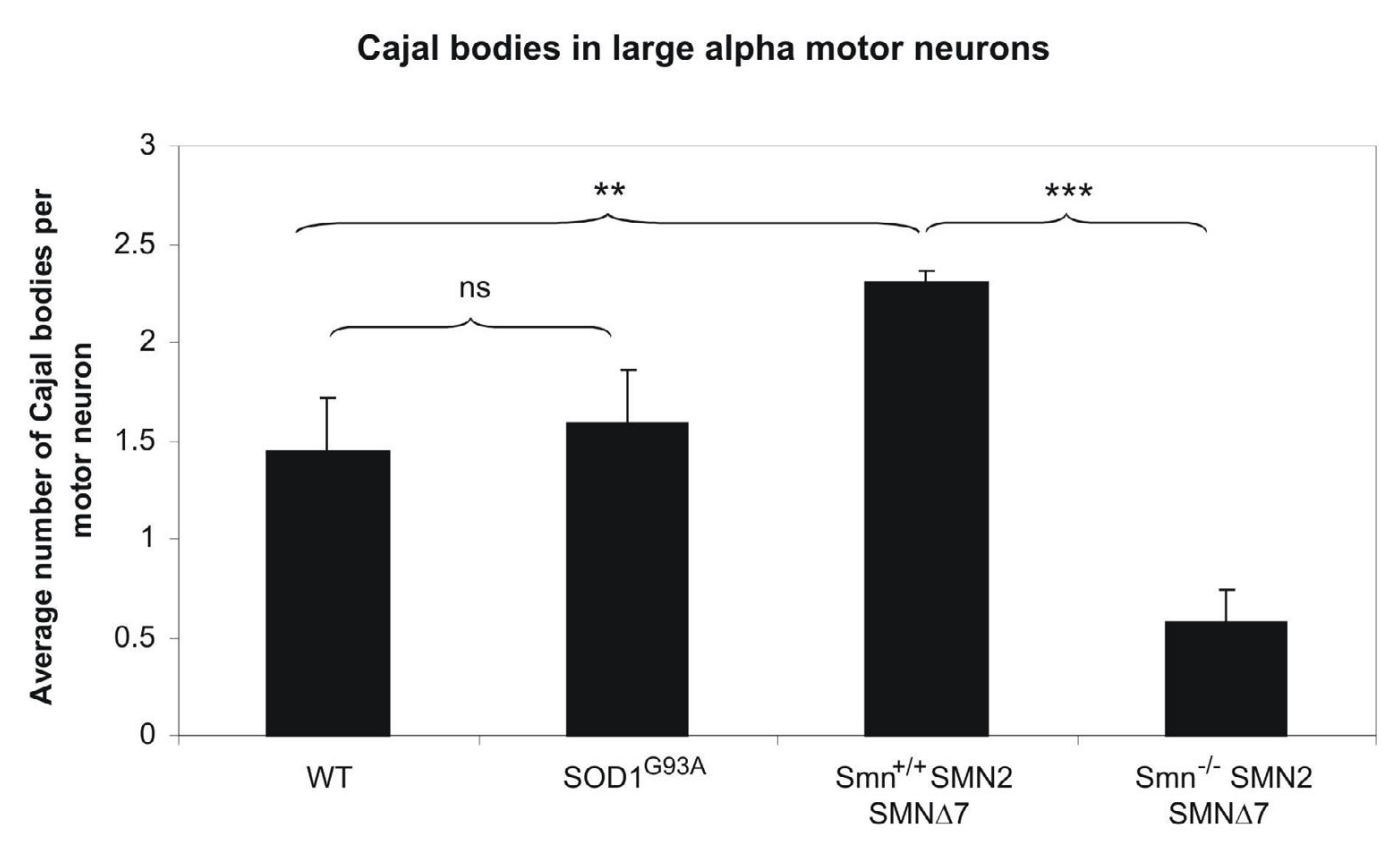
**Quantification of Cajal bodies per motor neuron nucleus for each genotype**. Unpaired two-tailed t-test, p = 0.0004 (***) and p = 0.002 (**). Error bars represent standard deviation.

**Figure 4:**
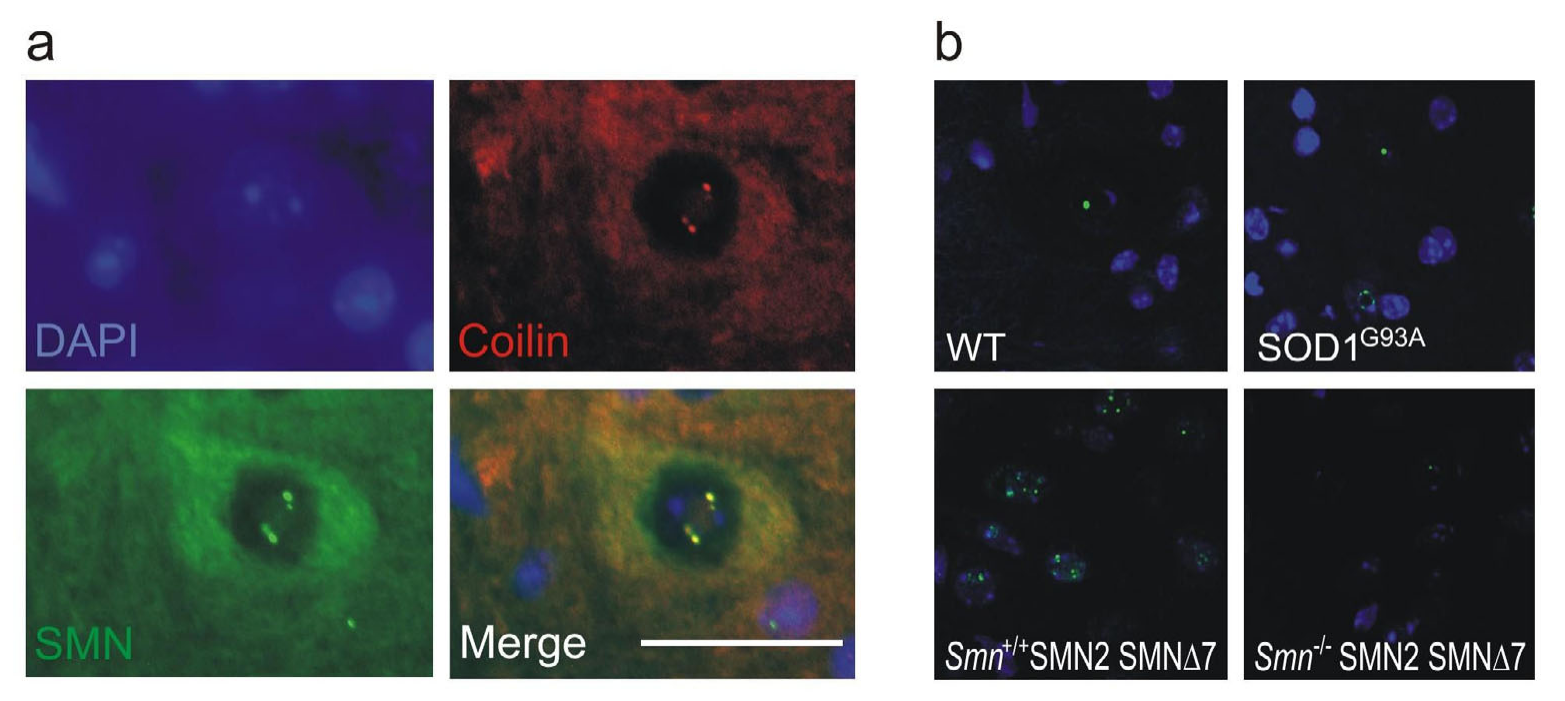
**(a) Wild-type mouse spinal cord sections stained for coilin and SMN.** Coilin co-localises with SMN in the motor neuron nucleus. (b) Coilin immunohistochemistry confirms reduction of Cajal bodies in the SMA mouse, but equal distribution between SOD1^G93A ^and WT animals. Scale bar = 20 μm.

Despite the lack of cytoplasmic TDP-43 pathology, its nuclear distribution may nonetheless be altered in motor neuron disease. In healthy motor neurons of WT mice, SMN localised to the cytoplasm and nuclear Cajal bodies (figure 2). In SMA animals, there was a marked reduction of cytoplasmic SMN staining as well as significant disruption of Cajal bodies (figures 2b, 3). To confirm that Cajal body depletion was not an artefact of the overall reduction in SMN immunoreactivity, we used coilin as an alternative marker of Cajal bodies. Coilin co-localised with SMN in motor neurons of WT animals (figure 4a), and was accordingly reduced in SMA animals (figure 4b).

There was no difference in SMN staining pattern or number of Cajal bodies between the SOD1^G93A ^and WT animals (figures 2, 3, 4). Of note, the Cajal body count was significantly higher in the Smn+/+;SMN2;SMNΔ7 animals than in the SOD1^G93A ^and WT animals. This difference might be due to the presence of increased levels of SMNΔ7 expressed from the SMNΔ7 and SMN2 transgenes in the SMA animals, but the different developmental state (13 days vs 4 months) or the different background strain could also play a role.

We found that the distribution of TDP-43 within the nucleus was unaltered both in SOD1^G93A ^and SMA animals when compared to their WT controls (figure 2). TDP-43 staining was granular, with sparing of the nucleolus, but showed discrete areas of high signal intensity suggesting the presence of distinct nuclear speckles. Although distinct accumulation of TDP-43 in discrete speckles is a consistent observation, it is unclear whether this has any functional significance. Furthermore, there was partial co-localisation with Cajal bodies in about 30% of cells examined (figure 2a).

**Figure 5:**
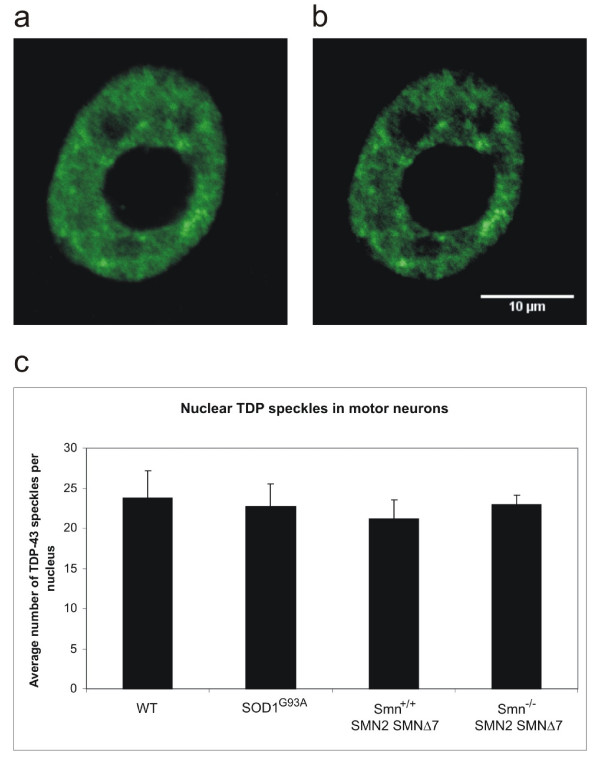
**Quantification of neuronal TDP-43 nuclear speckles in spinal cord sections.** Approximately 0.9 μm thick confocal section through representative motor neuron nucleus (a) and automatic setting of brightness and contrast in Image J (b). No significant difference in TDP-43 speckle number is obvious between genotypes (c).

To quantify the number of TDP-43 speckles per section, we used an auto-thresholding algorithm to separate areas of high signal from background granular staining. Comparison of TDP-43 nuclear speckles between WT and motor neuron disease animals revealed no significant difference (figure 5). Several limitations apply to this approach, however. First, we limited our analysis to motor neurons that were easily identifiable on the basis of their shape, possibly missing diseased cells with altered morphology. Likewise, we did not distinguish between cells that harboured ubiquitinated inclusions and those that did not. Thirdly, while quantification of TDP speckles is useful as an addition to qualitative assessment, the definition of what constitutes a TDP speckle is arbitrary and dependent on the intensity of the stain and the threshold setting during image acquisition.

### No change in TDP-43 expression or post-translational modification in SOD1^G93A ^or SMA animals

**Figure 6:**
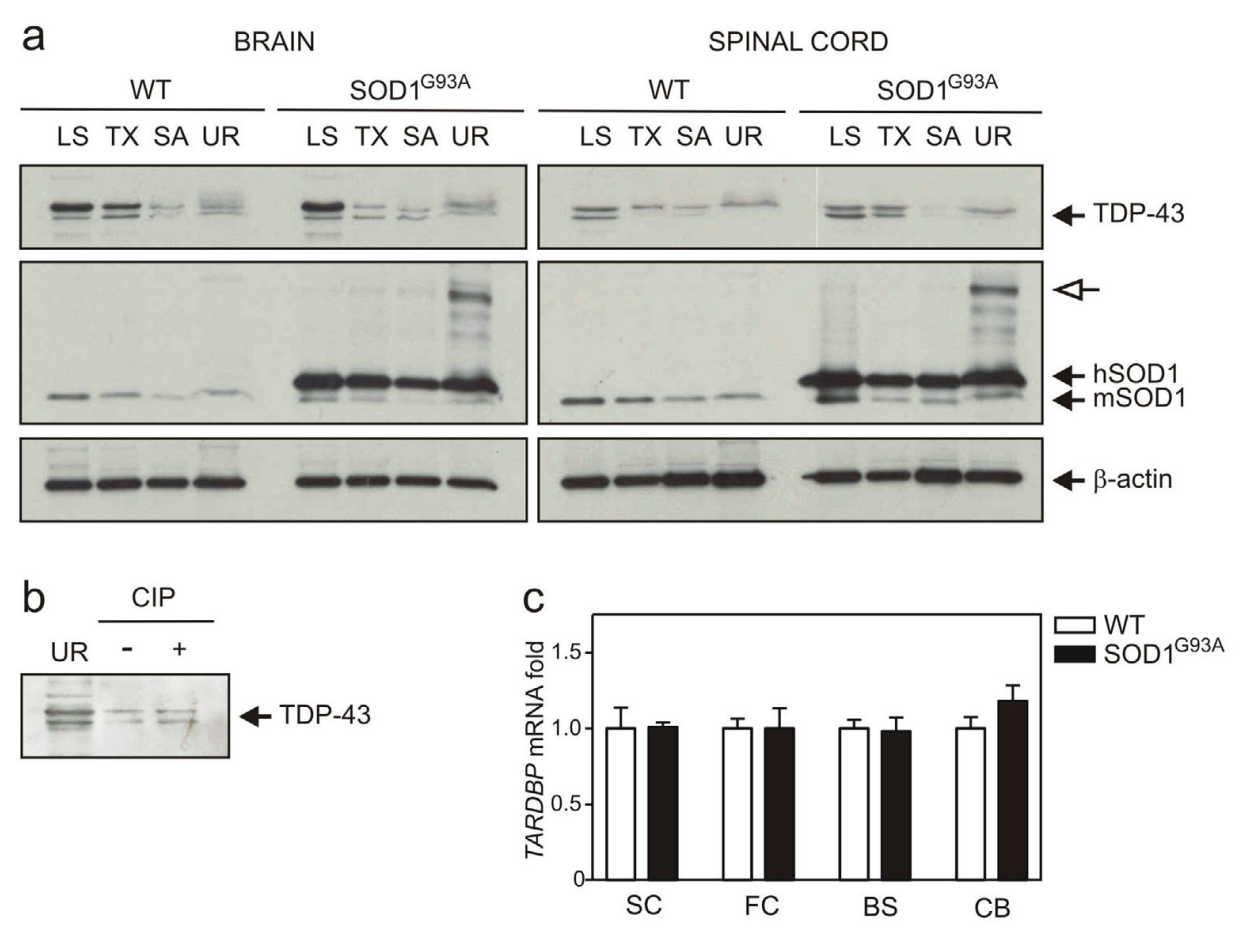
**TDP-43 protein and transcript levels in CNS of transgenic SOD1 G93A mice compared with age matched controls (a) Immunoblot analysis of brain and spinal cord extracts from wild-type (WT) and transgenic mice (SOD1^G93A^) at 4 months of age.** Open arrow, putative dimeric hSOD1 species. (b) Phosphatase treatment of representative brain urea extracts. (c) Quantitative RT-PCR analysis of brain regions and spinal cords from mice.

**Figure 7:**
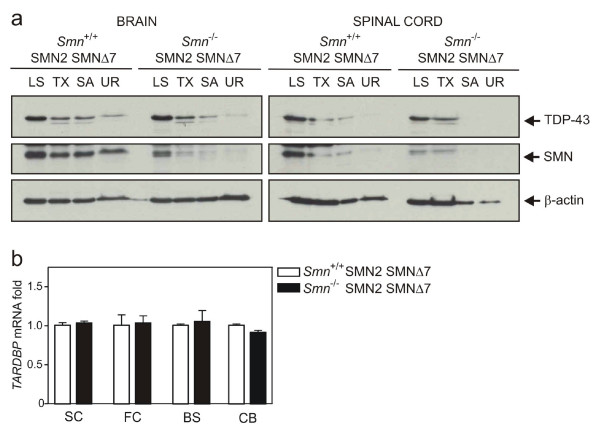
**TDP-43 protein and transcript levels in CNS of SMA mice (Smn^-/-^;SMN2;SMNΔ7) and age-matched littermates (a) Immunoblot analysis of brain and spinal cord extracts from wild-type (WT) and affected (SMA) mice at 13 days of age.** (b) Quantitative RT-PCR analysis of brain regions and spinal cords from mice.

In spite of evidence against TDP-43 redistribution or alteration of nuclear architecture, we additionally assessed whether abnormal TDP-43 biochemical species were present in SOD1^G93A ^or SMA animals. Brain and spinal cords were sequentially extracted in buffers of increasing ionic and detergent strength then analysed with immunoblotting. Full-length TDP-43 and a lower migrating species were detected in most fractions of transgenic mice, particularly abundant in LS extracts (figures 6a, 7a). However, the appearance and migration of TDP-43 was not altered compared to WT tissues. Endogenous and transgenic SOD1 was distributed across all fractions in ALS tissue with increased solubility of dimeric mutant SOD1 in urea (figure 6a). Likewise, endogenous SMN was found in all fractions in control brain, while doublet bands of putative SMN transgenic products mainly localised to LS extracts in SMA (figure 7a). To test for the presence of hyperphosphorylated TDP-43 in mice, urea extracts from CNS were dialysed and phosphatase treated (figure 6b). The persistence of an upper band suggests no phospho-TDP-43 species from this experiment.

Despite the lack of pathologic TDP-43 protein in SOD1^G93A ^or SMA mice, its transcription or mRNA stability may be regionally dysregulated in disease. Messenger RNA levels of TDP-43 were therefore quantified from three brain sub-regions and spinal cord using real-time PCR (figures 6c, 7b). TDP-43 transcript levels were similar across frontal cortex, brainstem, cerebellum and spinal cord in disease versus control mice.

## Discussion & conclusion

To our knowledge, this is the first study to examine the expression of TDP-43 at morphological and biochemical levels in mouse models of both ALS and SMA. It was prompted by two main questions: firstly, we wanted to confirm the previously reported lack of significant TDP-43 pathology in SOD1-mediated familial ALS in humans [17,23] and mouse models [20]. Independent confirmation of this observation is important, because the SOD1^G93A ^mouse model is the most commonly used model for human ALS and has informed many translational clinical trials. Understandably, the possibility that SOD-1-associated ALS may be pathogenetically distinct from sporadic ALS and most other forms of familial ALS, has caused controversy [24].

We found no evidence of TDP-43 mislocalisation or alteration of expression at the level of protein or mRNA in tissue extracts from endstage SOD1^G93A ^mice. In addition, the absence of C-terminally cleaved TDP-43 species in urea fractions of tissues, as well as lack of TDP-43 hyper-phosphorylation, is clear evidence that the SOD1^G93A ^mouse model does not share the characteristic pathological and biochemical hallmarks of TDP-43 pathology known from human sporadic ALS cases. This has important implications for the use of these mice in drug trials for ALS. While some drugs that showed efficacy in mouse drug trials, e.g. riluzole [25] have marginal efficiency in humans, this probably means that they exert a non-specific effect or act downstream from the disease-initiating pathological events. Clearly, a TDP-43 mouse model of ALS is needed to assess the degree of overlap between pathways of motor neuron degeneration in SOD1 and TDP-43 mediated disease for more efficient drug development. Although the core features of human sporadic TDP-43 pathology were not identified in our experimental setting of SOD1^G93A^-mediated disease, and at least one other study has reported similar findings in G37R and G85R SOD1 transgenic mouse models [20], it is conceivable that SOD1 and TDP-43 pathways interact at other, more subtle or as yet unidentified levels. For example, temporary alterations in TDP-43 expression or function over the course of SOD1-related ALS, cell-type specific changes (glial *versus *neuronal), or variations relating to specific SOD1 genotypes remain to be identified. Recent work identifying a covalently altered SOD1 species which is common to sALS and SOD1-fALS spinal cord suggests that it would be premature to conclude that there is no overlap between TDP-43 and SOD1 pathology [26].

The second aim of this study was to address the possibility that TDP-43 expression or nuclear architecture might be altered in SMA, which is characterised by low levels of SMN, a protein known to interact with TDP-43 in the nucleus [13]. While there is a distinct possibility that SMA is caused by loss of a neuron-specific, non-canonical function of SMN, e.g. in neuronal development, neurite outgrowth or axonal transport [27], there is mounting evidence that SMN deficiency leads to tissue-dependent differential defects in snRNP biogenesis and splicing [28,29]. This is in keeping with our finding of a marked reduction of Cajal bodies, which are sites of ribonuclear protein (RNP) maturation and snRNA modification [30,31], in motor neuron nuclei of SMA animals, and a potential site of SMN/TDP-43 interaction. The reduction in SMN protein and Cajal bodies was not accompanied by an alteration of TDP-43 mRNA or protein levels, nor a qualitative or quantitative change in nuclear distribution of TDP-43. This implies that SMA pathogenesis is not mediated by TDP-43, although, given the asymmetrical distribution of the two proteins, the abundance of TDP-43 in the nucleus might have masked small regional changes. A more rigorous examination of TDP-43 expression levels in laser-capture dissected anterior horn cells rather than whole tissue extracts may be needed to arrive at a definitive answer. In addition, there was no evidence that TDP-43 is up-regulated in compensation for SMN deficiency in the SMNΔ7 model of SMA.

While we were able to examine the effect of SMN deficiency on TDP-43 *in vivo *in the present study, we were unable to address the equally interesting question of what effect loss of nuclear TDP-43 might have on levels and distribution of SMN. In a study of frontotemporal lobar degeneration, Neumann and co-workers could not detect SMN in ubiquitinated, TDP-43 positive inclusions, and the SMN staining pattern using standard brightfield microscopy, as well as protein levels on a Western blot, were unaltered between disease and control cases [32]. However, it remains possible that loss of TDP-43 will lead to derangement of Cajal bodies, with resulting deficits in snRNP maturation. The fact that we did not observe any changes in SMN distribution in the SOD1^G93A ^model of ALS does not refute this hypothesis, as the TDP-43 distribution was, unlike in human sporadic ALS, unaltered. Further studies looking at SMN in TDP-43 positive ALS, using autopsy material or a model organism, are needed to assess this question.

## Authors' contributions

BT performed immunoblotting and real time PCR and drafted the manuscript. DB performed immunohistochemistry, confocal microscopy and drafted the manuscript. NP was responsible for animal breeding and preparation and carried out immunoblotting. JS performed immunhistochemistry and immunoblotting. KT and OA jointly conceived of the study, participated in its design and coordination and edited the final manuscript. All authors read and approved the final manuscript.
